# Three-Dimensional Pinecone-like Binder-Free Pt–TiO_2_ Nanorods on Ti Mesh Structures: Synthesis, Characterization and Electroactivity towards Ethanol Oxidation

**DOI:** 10.3390/molecules27061921

**Published:** 2022-03-16

**Authors:** Naser Mohammadi, Juan Carlos Abrego-Martinez, Mohamed Mohamedi

**Affiliations:** Énergie, Matériaux et Télécommunications (EMT), Institut National de la Recherche Scientifique (INRS), 1650 Boulevard Lionel Boulet, Varennes, QC J3X 1P7, Canada; naser.mohammadi@inrs.ca (N.M.); juan.abrego@inrs.ca (J.C.A.-M.)

**Keywords:** titanium dioxide, titanium mesh, platinum, hydrothermal method, pulsed laser deposition, ethanol electrooxidation

## Abstract

We report here the synthesis of binderless and template-less three-dimensional (3D) pinecone-shaped Pt/TiO_2_/Ti mesh structure. The TiO_2_ hydrothermally synthesized onto Ti mesh is composed of a mixture of flower-like nanorods and vertically aligned bar-shaped structures, whereas Pt film grown by pulsed laser deposition displays a smooth surface. XRD analyses reveal an average crystallite size of 41.4 nm and 68.5 nm of the TiO_2_ nanorods and Pt, respectively. In H_2_SO_4_ solution, the platinum oxide formation at the Pt/TiO_2_/Ti mesh electrode is 180 mV more negative than that at the Pt/Ti mesh electrode, indicating that TiO_2_ provides oxygeneous species at lower potentials, which will facilitate the removal of CO-like intermediates and accelerate an ethanol oxidation reaction (EOR). Indeed, the Pt/TiO_2_/Ti mesh catalyst exhibits current activity of 1.19 mA towards an EOR at a remarkably superior rate of 4.4 times that of the Pt/Ti mesh electrode (0.27 mA). Moreover, the presence of TiO_2_ as a support to Pt delivers a steady-state current of 2.1 mA, with an increment in durability of 6.6 times compared to Pt/Ti mesh (0.32 mA). Pt is chosen here as a benchmark catalyst and we believe that with catalysts that perform better than Pt, such 3D pinecone structures can be useful for a variety of catalytic or photoelectrochemical reactions.

## 1. Introduction

There is a growing need for lightweight, higher efficiency, cheap, durable and safe energy storage and conversion devices to meet special needs for next-generation high-performance portable electronic devices. Current research is focusing on flexible and wearable electronic devices such as roll-up display, light-emitting diodes, smart watches, fitness-tracking and implantable sensors that can monitor blood pressure and other health metrics [1]. Such devices are being developed rapidly and essentially require low power source systems with flexible electrodes, separators and substrates to accomplish all-in-one flexible systems. Several types of lightweight power sources are being developed worldwide, including Li-ion batteries, supercapacitors, solar cells, fuel cells and biofuel cells [2,3,4,5,6].

Generally, traditional electrodes used in electrochemical power sources (EPSs) are composed of mixtures of active materials, polymeric binders and conductivity enhancers, like carbon black, graphite and carbon nanotubes, which are ink-coated over metal current collectors such as copper, aluminum or carbon paper. The additives, the metal collector elements are not only unsuitable for flexibility but add extra-weight to the EPS device. Hence, the ability to eliminate such additives and fabricate a binder and additive-free, flexible electrode would signify notable advancement for high-performance flexible EPSs. A flexible electrode could be either an electroactive material with inherent flexibility, or a composite catalyst layer built on a flexible substrate.

Metallic grids or meshes based on Ti as flexible current collectors are receiving a lot of attention due to their excellent flexibility, conductivity, and three-dimensional (3D) structures that can host active materials. A few examples of using Ti mesh in electrochemical technology include fabrication of high performance Pt/Ti counter electrodes on Ti mesh for flexible large-area dye-sensitized solar cells [7], TiO_2_ nanotubes on Ti mesh electrodes for improved photoelectrochemical reaction [8], TiO_2_ nanotube arrays formed on Ti meshes for flexible dye-sensitized solar cells [9], TiO_2_ nanotubes grown on Ti grid as an anode for Li-ion microbatteries [10], MnO_2_-modified TiN nanotube arrays on Ti mesh for flexible supercapacitor electrodes [11] and electrochemical glucose sensors using ternary NiCoP nanosheet arrays deposited on a Ti mesh [12].

In addition to the fact that TiO_2_ is not expensive to synthesize, non-toxic, possesses high mechanical and corrosion resistance, chemical stability in both alkaline, acidic, and oxidative environments; TiO_2_ has been investigated as a substitute catalyst support to carbon in fuel cells such as H_2_/O_2_ [13,14,15], direct methanol fuel cells (DMFC) [16,17,18,19] and microbial fuel cells [20,21].

In this work, we report for the first time the synthesis of original 3D pinecone-like Pt–TiO_2_ nanorods on Ti mesh electrodes. TiO_2_ arrays are grown via a hydrothermal technique on Ti mesh, whereas Pt film is grown by pulsed laser deposition (PLD) onto the TiO_2_/Ti mesh. For comparison, Pt is also synthesized by PLD on bare Ti mesh. The structural properties of these materials are studied with scanning electron microscopy (SEM), X-ray diffraction (XRD) and X-ray photoelectron spectroscopy (XPS). The electron transfer properties of the Pt/TiO_2_/Ti mesh are assessed for the ethanol oxidation reaction (EOR) with linear scan voltammetry (LSV). The EOR was chosen first, because of its importance for direct ethanol fuel cell (DEFC) technology and also, because we have been actively studying the EOR for years. Pt is selected here as a model catalyst because of its well-known behavior towards EOR. Finally, the durability of the synthesized Pt/TiO_2_/Ti mesh compared with Pt/Ti mesh is studied with long-term chronoamperometry (CA).

## 2. Results and Discussion

### 2.1. Materials Characterization

**Figure 1:**
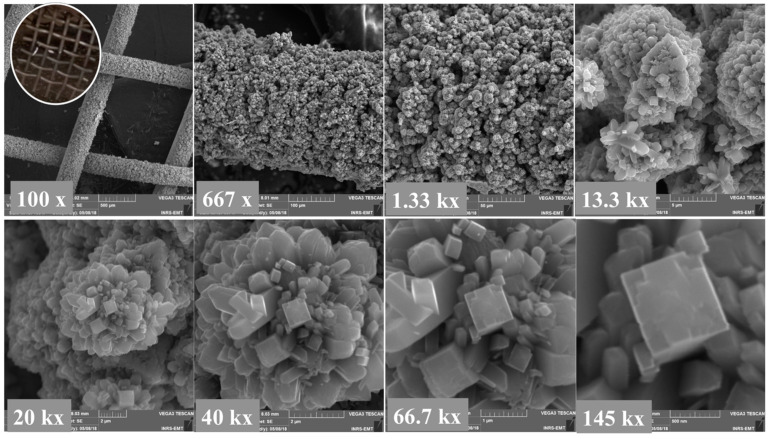
SEM images at different magnifications of hydrothermally-grown TiO_2_ on Ti mesh. Inset at the top left figure is a photograph of TiO_2_/Ti mesh.

The photograph and SEM images of the pristine Ti mesh are shown in Figure S1 (Supplementary Materials) for comparison. Figure 1 reports SEM micrographs at increasing magnifications of the TiO_2_ grown by the hydrothermal technique on Ti mesh. As can be seen from SEM the images, the Ti mesh was evenly coated with TiO_2_. Higher magnifications show that the as-prepared TiO_2_ is composed of a mixture of flower-like nanorods and vertically aligned bar-shaped structures.

**Figure 2:**
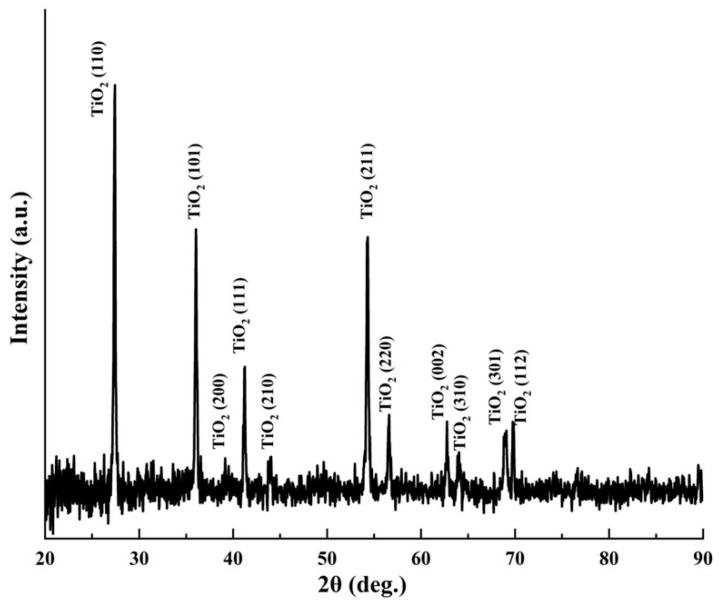
XRD analysis of TiO_2_ grown on Ti mesh via the hydrothermal method.

The XRD crystalline structure corresponding to the TiO_2_/Ti mesh is reported in Figure 2. The peaks relate to rutile TiO_2_ (JCPDS 21-1276). The highest peak TiO_2_ (110) was employed to calculate the average crystallite size (*L*). This was done using the Debye–Scherrer equation: *L* = 0.89 *λ*/*β* cos *θ*, with *λ* corresponding to the wavelength of 1.5406 Å, *β* to the full-width at half-maximum (FWHM) and *θ* is the Bragg angle. Accordingly, the average crystallite size of the TiO_2_ nanorods was estimated to be 414 Å.

**Figure 3:**
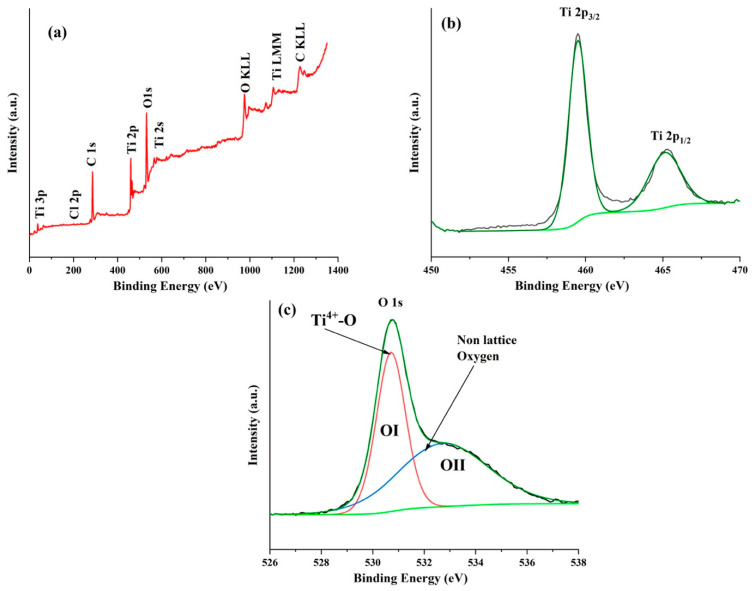
XPS analyses of TiO_2_/Ti mesh. (**a**) Survey scan, (**b**) high-resolution XPS spectrum of Ti 2p core level and (**c**) high-resolution XPS spectrum O 1s core level.

Figure 3 shows the XPS spectra of the TiO_2_/Ti mesh structure. The survey spectrum (Figure 3a) contains the most prominent lines of Ti 2p, O 1s and C 1s at standard positions. Carbon contamination is practically impossible to avoid. Fortunately, it is usually restricted to the film-air interface. Figure 3b,c reveal, respectively, the high-resolution XPS Ti 2p and O 1s peaks at the surface of TiO_2_/Ti mesh. The positions of the Ti 2p_1/2_ and Ti 2p_3/2_ separated by 5.60 to 5.64 eV demonstrating by that Ti was in the Ti^4+^ state at the surface [22,23,24]. The O 1s peak is asymmetrical, which signifies that oxygen appears in two different chemical states at least. The O 1s spectrum (Figure 3c) could be deconvoluted into two contributions that are OI and OII. The first contribution (OI) occurring within binding energies of 529.9~530 eV is ascribed the normal lattice sites occupied by oxygen in the TiO_2_ structure. The second contribution (OII) appearing at 531~532 eV is ascribed to non-lattice oxygen [25]. This high binding energy component has been assigned to oxygen bonded to Ti^+3^ (O–Ti^3+^) or to hydroxyl species that are simply created at the surface of oxide films [26]. By considering the relative areas related to the main O 1s component (linked principally to Ti^4+^–O bonds) and the relative area of the main Ti 2p contribution, we obtain an [O]/[Ti] ratio of 3.8. Meng et al. have studied the porosities of titanium oxide films prepared by d.c. reactive magnetron sputtering at different oxygen partial and total pressures [27]. They have ascribed the presence of the non-lattice O component to porosity, as a result of the moisture that accrues mainly in the pores or void between the TiO_2_ columns of the material, which might be responsible for the deviation from stoichiometry. The same authors have employed the ratio of O_II_**/**(O_I_ + O_II_) as a measure of the porosity. In our case, we found the O_II_**/**(O_I_ + O_II_) ratio equal to 0.44, which is slightly higher than 0.38 obtained by Meng et al. for porous TiO_2_ film that had a higher O-H bonding component. Therefore, it may be assumed that our synthesized TiO_2_ arrays display porosity higher than the stoichiometric films.

### 2.2. Characterization of Pt/TiO_2_/Ti Mesh

**Figure 4:**
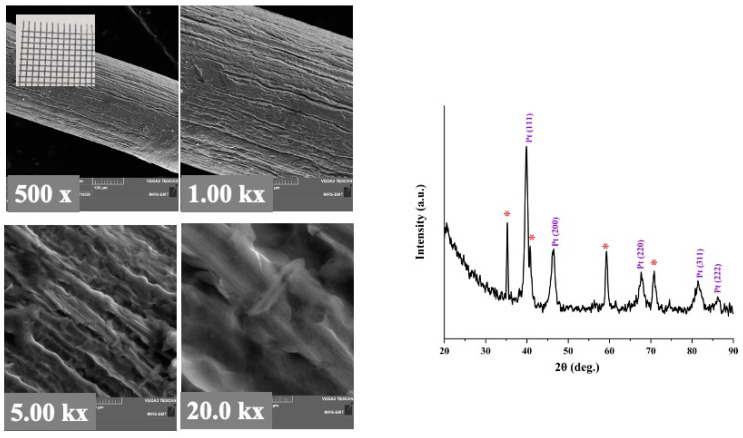
SEM micrographs at increasing magnifications of Pt grown by PLD on Ti mesh. Inset at the top left of the figure is a photograph of Pt/Ti mesh. The figure on the right side is the XRD analysis of Pt/Ti mesh. (*) Corresponds to the TiO_2_ substrate.

SEM images of the Pt/Ti mesh structure revealed that the surface morphology of the Pt film is very smooth (Figure 4). The XRD indexation (Figure 4, right side) of Pt peaks is in agreement with (111), (200), (220), (311) and (222) planes, respectively, of a face centered cubic (fcc) structure (JCPDS PDF No. 04-0802). The most intensive diffraction peak Pt (111) was selected to calculate the lattice constant (*a*) and the average crystallite size of Pt by Bragg’s law and Debye–Scherrer equation, respectively. The average crystallite size of Pt deposited Ti mesh was found to be 932 Å. On the other hand, the lattice constant was calculated to be close to 3.924 Å, which is close to the theoretical value of 3.923 Å.

**Figure 5:**
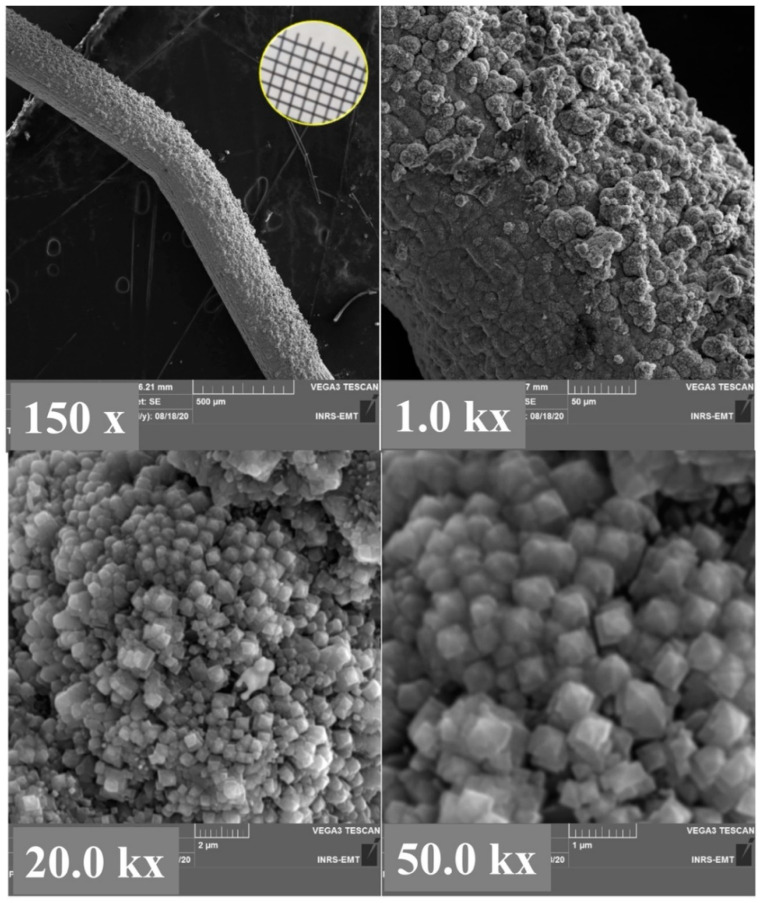
SEM micrographs at increasing magnifications of Pt grown by PLD on a TiO_2_/Ti mesh structure. Inset at the top left of the figure is a photograph of Pt/TiO_2_/Ti mesh.

**Figure 6:**
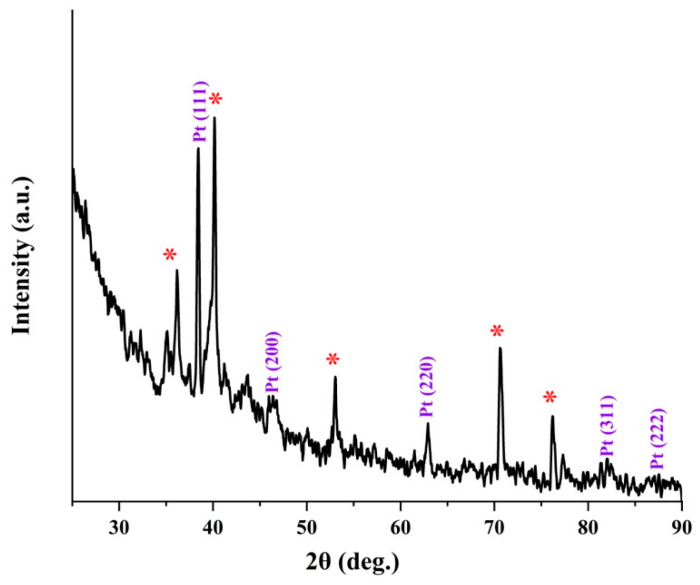
XRD analysis of Pt grown on TiO_2_/Ti mesh. (*) Corresponds to the TiO_2_ substrate.

The morphology of Pt films deposited on the TiO_2_ arrays is unlike Pt synthesized on Ti mesh. Figure 5, at higher magnification, shows that the Pt/TiO_2_ resembles to 3D pinecone-shaped structure. The lattice parameter and the average crystallite size calculated from XRD of Figure 6 were found to be 3.885 Å and 685 Å, respectively. Table 1, resumes the various XRD characteristics of the Ti mesh, TiO_2_/Ti mesh, Pt/Ti mesh and Pt/TiO_2_/Ti mesh. From the table, it can be seen that the average crystallite size of Pt is smaller than that of Pt grown on Ti mesh, suggesting that TiO_2_ enhanced the distribution quality of the Pt nanoparticles. It can also be observed that the lattice parameter of Pt synthesized on TiO_2_ is lesser than that of Pt produced on Ti mesh. This might exhibit a size-induced lattice contraction in the as-prepared state with respect to bulk Pt. The interaction of oxides with metallic nanostructures has been extensively investigated with particular emphasis on the effect of surface oxygen vacancies [28]. Oxygen vacancies at the metal support interface are recognized to induce charge transfer from the oxide to the metal easing the binding of the metal to the oxide. It has been reported that Pt is strongly adsorbed at oxygen vacancy sites in TiO_2_ [29]. It has previously been discussed that lattice strain can dramatically affect electrochemical activity [30,31,32]. Hence it is anticipated that the support-induced lattice strain observed in Pt/TiO_2_ could potentially enhance electrocatalytic activity, compared with Pt/Ti mesh.

**Figure 7:**
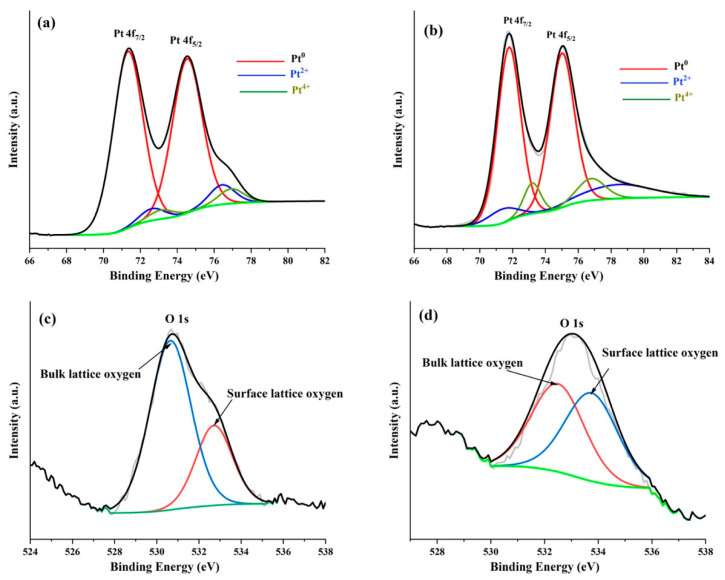
High-resolution XPS spectra of Pt 4f core level and O 1s core level. (**a**,**c**) Pt/Ti mesh. (**b**,**d**) Pt/TiO_2_/Ti mesh.

The XPS survey spectrum of the Pt/TiO_2_ material shown in Figure S2 (Supplementary Materials) reveals that the surface exclusively consists of Pt, O and C elements. No Ti metal is detected indicating that the Pt film homogenously coats the underneath TiO_2_ nanorods. Figure 7a,b report the high-resolution Pt 4f core-level spectrum at the Pt/Ti mesh and Pt/TiO_2_/Ti mesh, respectively. The spectra show that Pt exists in the form of at least three oxidation states. The spectra were indeed adequately deconvoluted into three overlapping curves assigned to Pt^0^, Pt^2+^ and Pt^4+^ species. Table 2 shows the binding energy (*BE*) and relative amount of these three species assessed from the relative area of the integrated peak intensities. The peak positions of Pt^0^, Pt^2+^ and Pt^4+^ are in agreement with the values found in the literature [33,34]. The shift toward higher *BE* values compared to literature values (71.0 eV) is ascribed to the metal−support interaction and to small Pt nanoparticle sizes [35,36]. This positive shift may also imply metal−support interactions between TiO_2_ and Pt as observed with XRD analyses. This interaction can change the electronic properties of Pt by increasing the Pt *d*-vacancy via electronic donation to Lewis acid centers such as Ti^x+^ at the Pt/TiO_2_ interface [37,38,39].

From Table 2, it can be noted that in the Pt/Ti mesh, Pt^0^ is widely distributed on the surface with 87.7 at% followed by slight relative concentrations (<7 at%) of Pt^2+^ and Pt^4+^. It should be noted that for Pt deposition on the Ti mesh, the former has not been etched on purpose, in order to better assess the effect of TiO_2_ morphology (layer vs. nanorods) on the electrochemical performance. Hence, the oxygen is due to the native TiO_2_ layer present on the surface of the mesh. On the other hand, the concentration of Pt^0^ decreased while those of Pt^2+^ and Pt^4+^ increased at the Pt/TiO_2_ sample. From Table 2, compared to the Pt/Ti mesh, it was noticed that in the Pt/TiO_2_/Ti mesh the position of Pt^0^ shifted by 0.45 eV toward higher *BE*s while the position of Pt^2+^ moved by 1.26 eV toward lower *BE*s. These shifts can be explained by different sizes of Pt particles and different degrees of interaction with the TiO_2_ support, signifying a strong interaction between the TiO_2_ nanorods and the Pt film above. Other researchers have observed Pt/TiO_2_ composites exhibiting ionized platinum, which was also ascribed to the strong interaction between Pt and the TiO_2_ support [37,38,40]. This behavior can be assumed to the presence of oxygen vacancies at the TiO_2_ support interface. The Pt^4+^ (PtO_2_) is the result of Pt cations replacing those of Ti in the TiO_2_ lattice, and the Pt atoms at the surface creating Pt^2+^ species. The O 1s core level peaks for the Pt/Ti mesh and Pt/TiO_2_/Ti mesh materials are shown in Figure 7c,d, respectively. A simple visual inspection of the O1s peak showed that it was wide and asymmetrical (Figure 7c,d). Therefore, the peak could be deconvoluted in two peaks. The resulting parameters are reported in Table 2. In this case, the OI element is ascribed to the bulk lattice oxygen, whereas the OII component is attributed to the surface lattice oxygen [41].

### 2.3. Electrochemical Characterization

**Figure 8:**
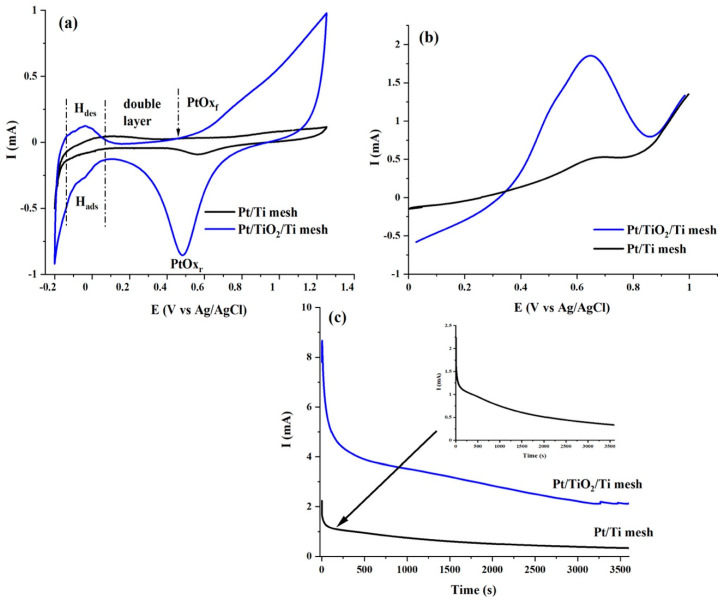
Electrochemical studies (**a**) Cyclic voltammetry in 0.5 M H_2_SO_4_-argon purged solution with a potential scan rate of 50 mV s^−1^. (**b**) Linear scan voltammetry in 0.5 M H_2_SO_4_ + 1 M C_2_H_5_OH solution with a potential scan rate of 5 mV s^−1^. (**c**) Chronoamperometry in 0.5 M H_2_SO_4_ + 1 M C_2_H_5_OH solution.

Figure 8a recorded with 50 mV s^−1^ compares CVs of Pt/Ti mesh and Pt/TiO_2_/Ti mesh electrodes in 0.5 M H_2_SO_4_ deaerated solution. It has to be reiterated that both electrodes contained a similar amount of Pt (0.120 mg cm^−2^). At the Pt/TiO_2_/Ti mesh electrode, the CV contained the classical features of hydrogen atom adsorption (H_ads_) and hydrogen atom desorption (H_des_) peaks from −0.2 to 0.1 V [42,43,44,45]. On the other hand, the H_ads_/H_des_ features were ill-defined at the Pt/Ti mesh electrode. In addition, the current of the H_ads_/H_des_ peaks at the Pt/TiO_2_/Ti mesh were distinctively greater than those delivered by the Pt/Ti mesh, which clearly indicates the greater surface area at the former electrode [45]. This also means that Pt/TiO_2_ has a larger surface area than the Pt/Ti mesh. The larger surface area signifies smaller particle size, confirming that TiO_2_ improved the dispersion and utilization of the Pt nanoparticles [46], which is in line with the XRD analyses (Table 1). The Pt oxide formation (PtOx_f_) at the Pt/Ti mesh starts at 0.74 V whereas its equivalent reduction (PtOx_r_) peak potential takes place at 0.56 V vs. Ag/AgCl. On the other hand, PtOx_f_ and PtOx_r_ were located at 0.44 V and 0.48 V, respectively at the Pt/TiO_2_/Ti mesh. This implies that TiO_2_ can provide oxygeneous species at lower potentials, which will facilitate the removal of CO-like intermediates and accelerate EOR.

Subsequently, the effect of the TiO_2_ arrays on the electrocatalytic behaviour of Pt was investigated towards electrooxidation of ethanol. Figure 8b shows comparative LSVs obtained in 0.5 M H_2_SO_4_ + 1 M C_2_H_5_OH solution at 5 mV s^−1^ (quasi-steady state) at Pt/Ti mesh and Pt/TiO_2_/Ti mesh electrodes. Forward and backward CVs in the 0.5 M H_2_SO_4_ + 1 M C_2_H_5_OH solution are shown in Figure S3. The LSVs at both electrodes showed characteristic EOR waves in accordance with the literature [47,48,49]. The onset potential, *E*_onset_, peak potential (*E*_p_), *I*@0.50 V, and peak current *I*_p_ values extracted from LSVs of Figure 8b are reported in Table 3. *E*_onset_ is a criterion that provides knowledge about the kinetics of an electrochemical reaction and is identified here as the value at which a current begins for the electrooxidation of ethanol. *I*@0.50 V is an experimental condition near to the projected functioning potential of DEFCs devices and allows comparison of the progress of the EOR catalytic activity using different electrocatalysts. Hence from Table 3, it can be observed that *E*_onset_ and *E*_p_ are not significantly different at both electrodes. However, Pt/TiO_2_/Ti mesh demonstrated the best catalytic activity toward EOR in terms *I*_p_ of 1.85 mA which is remarkably 3.5 times greater than the *I*_p_ delivered by Pt/Ti mesh. A further outstanding performance of the Pt/TiO_2_/Ti mesh is obviously its value of *I*@0.5 V, which is 4.4 times greater than the Pt/Ti mesh.

Chronoamperometric (CA) experiments were performed to examine the electrodes durability. Figure 8c shows the current-time (*I*-*t*) curves of the Pt/Ti mesh and Pt/TiO_2_/Ti mesh for EOR at 0.6 V upon 3600 s testing. It can be seen that in both CA profiles, the current increased abruptly, then decreased and ultimately attained quasi-steady-state behavior. In Figure 8c, one can observe that the current decay for the EOR on the Pt/TiO_2_/Ti mesh catalyst is significantly slower than that on the bare Pt/Ti catalyst. The first current increase is due to the double-layer charging effect, whereas the initial decay was caused by the rapid increase of the surface coverage by intermediate species, such as adsorbed CO during EOR [48]. After 3600 s, the quasi steady-state current density (*I*_ss_) at the Pt/TiO_2_/Ti mesh was 6.6 times greater than that of Pt/Ti mesh (Table 3).

In summary, it is clear that incorporating TiO_2_ flower-like nanorods and vertically aligned bar-shaped structures as a supporting material considerably increased the electrochemical activity of the Pt catalyst. Therefore, it can be suggested that the Pt/TiO_2_/CP catalyst offers a higher Pt utilization than the unsupported Pt/Ti mesh catalyst.

## 3. Materials and Methods

### 3.1. Growth of TiO_2_ Arrays onto Ti Mesh

Titanium meshes of 1.5 cm × 2 cm with 0.5 mm thickness were placed in an ultrasonic bath containing acetone and washed for 20 min. After rinsing with water and drying in air, the titanium meshes were subjected to chemical etching in a solution containing 15 mL of HCl (18 wt%) solution. The chemical etching was conducted at a temperature of 80 °C and a duration of 15 min. This operation was necessary to remove the native TiO_2_ layer. Afterward, the Ti meshes were placed inside a Teflon stainless steel autoclave (23 mL, Parr Instrument, Moline, IL, USA) containing 10 mL of 0.6 M of HCl aqueous solution. The hydrothermal synthesis was conducted at 180 °C and lasted 10 h. The synthesis condition effects on the microstructure of the obtained materials can be found in our previous publication [50].

### 3.2. Growth of Pt onto Ti Mesh and TiO_2_ Arrays

Platinum films were deposited onto Ti mesh and TiO_2_/Ti mesh substrates at room temperature by PLD method employing pure Pt target (99.99%, Kurt J. Lesker Co, Jefferson Hills, PA, USA). Details on the operating principle of the PLD are reported elsewhere [51,52]. The deposition conditions were: 50 kp laser pulses, 2 Torr of helium, KrF excimer laser (*λ* = 248 nm), pulse width of 17 ns, repetition rate of 50 Hz, 4 Joules per cm^2^ as the laser fluence and 5 cm as the distance between the substrate (Ti or TiO_2_) and the Pt target. Prior to every deposition of Pt, evacuation of the PLD chamber was done at 4 × 10^−5^ Torr by a turbo pump. The amount of the deposited Pt assessed with neutron activation analysis was 120 μg cm^−2^. The PLD deposition parameters were optimal and reported in our previous publications [53,54].

### 3.3. Materials Characterization

An SEM (TESCAN, LYRA3) operated at 20 kV was used to analyze the surface morphology of the synthesized materials. The crystalline structure of all samples was determined by XRD using a Bruker D8 Advance diffractometer equipped with a Cu K*α* source (*λ* = 1.5406 Å). The tube current was 40 mA with a tube voltage of 40 kV. Diffractograms were acquired with an acquisition time of 5 s per step in the Grazing Incidence Diffraction (GID) scan mode using an incident angle of 3° and a 2*θ* angular of 0.04° step size. XPS analysis was conducted to examine the concentration of the elements and their valence states at the surface of the samples with a VG Escalab 220i-XL outfitted with an Al K*α* source (1486.6 eV). 10 kV, 20 mA and pass energy of the analyzer 20 eV were the conditions that operated the anode. Survey spectra were first recorded from 0 to1350 eV. Afterwards, higher resolution multiplex scans (Ti 2p, Pt 4f, C1 s and O 1s core levels) were acquired. CasaXPS software (Casa Software Ltd, Teignmouth, UK.) was employed to analyze and quantify the elements by fitting the core level spectra to a Shirley background exclusion. The metallic components of the Pt 4f and Ti 2p regions were fitted using a Gaussian/Lorentzian asymmetrically modified line shape, and a Gaussian/Lorentzian line shape was used to fit the other components. The C 1s core level located at 284.6 eV, stemming from hydrocarbon impurities present at the surface of the samples, was employed as an internal reference. All XPS spectra were readjusted with regard to the C 1s core level of accidental carbon impurity.

### 3.4. Electrochemical Experiments

Ethanol (100% purity) and sulfuric acid (H_2_SO_4_, 96%) were acquired from Commercial Alcohols Inc. (Toronto, ON, Canada) and Agros Organics (Fisher Scientific, Mississauga, ON, Canada), respectively. The reactants were used as received. The electrochemical properties were studied by voltammetry or LSV. The electrolytic solution was either 0.5 M H_2_SO_4_ or 1 M C_2_H_5_OH + 0.5 M H_2_SO_4_. The 3-electrode cell contained Ag/AgCl (4 M NaCl) that acted as a reference electrode, a platinum coil as an auxiliary electrode and Pt/Ti mesh or Pt/TiO_2_ arrays/Ti mesh as working electrodes. In this paper, the potentials are reported against Ag/AgCl. Before commencing each electrochemical experiment, argon was bubbled through the electrolytic solution for 30 min to remove dissolved oxygen. Then the surface of the Pt/Ti mesh and Pt/TiO_2_/Ti mesh were subjected to electrochemical cleaning and activation in 0.5 M H_2_SO_4_ by multicycling voltammetry from −0.2 V to 1.3 V at 50 mV s^−1^ potential scan rate until a steady state voltammogram was reached. EOR experiments were performed with LSV using a mixture of 1 M C_2_H_5_OH and 0.5 M H_2_SO_4_ within 0 V to 1.0 V at 5 mV s^−1^. The Ti meshes in all cases had the same geometric size. Electrochemical measurements were conducted at ambient temperature using an Autolab, PGSTAT 20.

## 4. Conclusions

TiO_2_ arrays were successfully synthesized directly on the Ti mesh by a hydrothermal method in acidic medium. The hydrothermal method does not necessitate the utilization of templates, it is easily scalable, cheap and environmentally benign. SEM observations revealed that as-prepared TiO_2_ is constituted of a mixture of flower-like nanorods and vertically aligned bar-shaped structures corresponding to rutile phase, as identified by XRD. By means of XPS, the [O]/[Ti] atomic ratio was found to 3.8. This deviation from stoichiometry is ascribed to porosity that permits moisture to accrue between the voids of the perpendicularly arranged TiO_2_ bars or within the arranged flower-like TiO_2_ nanorods.

Afterwards, Pt catalyst as a benchmark catalyst was deposited by PLD onto synthesized TiO_2_ structures in order to assess their catalytic supporting properties. SEM imaging revealed an interesting 3D pinecone-shaped Pt/TiO_2_ structure. XRD analysis showed that the crystallite size of Pt in Pt/TiO_2_ was smaller than that in Pt/Ti mesh, which demonstrates that the TiO_2_ support enhances the dispersion quality of Pt nanoparticles. Furthermore, XPS analysis confirmed the strong interaction between Pt and the TiO_2_ support, which induces ionized platinum (Pt^2+^ and Pt^4+^).

Notwithstanding having a similar amount of Pt, the three-dimensional pinecone-shaped Pt/TiO_2_ structure exhibited current catalytic activity towards EOR at a remarkably greater rate of 4.4 times more than unsupported Pt. Moreover, the presence TiO_2_ as support enables 6.6 times increased current durability relative to the Pt/Ti mesh. As mentioned in this work, Pt was chosen here as a model catalyst and we believe that with catalysts that perform better than platinum such 3D mesh architectured electrodes are promising not only for fuel cells in general but can be useful for a variety of catalytic or photoelectrochemical reactions for other catalysts.

## Figures and Tables

**Table 1:** XRD characteristics of the Ti mesh, TiO_2_/Ti mesh, Pt/Ti mesh and Pt/TiO_2_/Ti mesh.

	Ti Mesh	TiO_2_/Ti Mesh	Pt/Ti Mesh		Pt/TiO_2_	
	Ti	Ti	Ti	Pt	Ti	Pt
Lattice	a = 2.953	a = 2.957	a = 2.951	a = 3.924	a = 2.960	a = 2.953
constant (Å)	c = 4.687	c = 4.599	c = 4.682		c = 4.598	c = 4.687
L (Å)	607.1	581.7	551.8	932.3	414.6	685.5

**Table 2:** XPS fitting parameters from the core-level spectra of Pt 4f and O 1s.

Sample	Pt^0^		Pt^2+^		Pt^4+^			O 1s	
	*BE* (eV)	at%	*BE* (eV)	at%	*BE* (eV)	at%	Pt^2^/Pt^4+^	O_I_	O_II_
Pt/Ti mesh	71.33	87.73	72.63	7.01	73.13	5.25	1.34	530.64 (70.5)	532.71 (29.5)
Pt/TiO_2_	71.78	75.90	71.37	13.15	73.20	10.95	1.20	532.50 (50)	533.75 (50.0)

Note: Numbers between brackets are species surface percentage.

**Table 3:** Comparative main electrochemical performance parameters.

	EOR				Durability
	*E*_onset_ (V)	*E*_p_ (V)	*I*_p_ (mA)	*I@*0.5 V (mA)	*I*_ss_ (mA)
Pt/Ti mesh	0.26	0.67	0.53	0.27	0.32
Pt/TiO_2_/Ti mesh	0.30	0.65	1.85	1.19	2.1

## Supplementary Materials

The following supporting information can be downloaded online https://www.mdpi.com/article/10.3390/molecules27061921/s1, Figure S1: SEM micrographs at increasing magnifications of pristine Ti mesh. Figure S2: XPS survey scan of Pt film grown onto TiO_2_ nanorods; Figure S3: Cyclic voltammetry in 0.5 M H_2_SO_4_+ 1 M C_2_H_5_OH solution with a potential scan rate of 5 mV s^−1^.

## Sample Availability

Samples of the compounds are not available from the authors.
