# Supplementary material for: Three-Dimensional Pinecone-like Binder-Free Pt–TiO2 Nanorods on Ti Mesh Structures: Synthesis, Characterization and Electroactivity towards Ethanol Oxidation
Source: Molecules. 2022 Mar 16;27(6):1921. doi: 10.3390/molecules27061921 (PMC8955681; doi:10.3390/molecules27061921)
Supplement: Supplementary file 1 [file molecules-27-01921-s001.zip › molecules-1576098-supplementary.pdf]

Supplementary Materials

Three-Dimensional Pinecone-like Binder-Free Pt–TiO_2 Nanorods on Ti Mesh Structures: Synthesis, Characterization and Electroactivity towards Ethanol Oxidation

Naser Mohammadi, Juan Carlos Abrego-Martinez and Mohamed Mohamedi *

Énergie, Matériaux et Télécommunications (EMT), Institut National de la Recherche Scientifique (INRS),
1650 Boulevard Lionel Boulet, Varennes, QC J3X 1P7, Canada;
naser.mohammadi@inrs.ca (N.M.); juan.abrego@inrs.ca (J.C.A.-M.)
* Correspondence: Mohamed.Mohamedi@inrs.ca

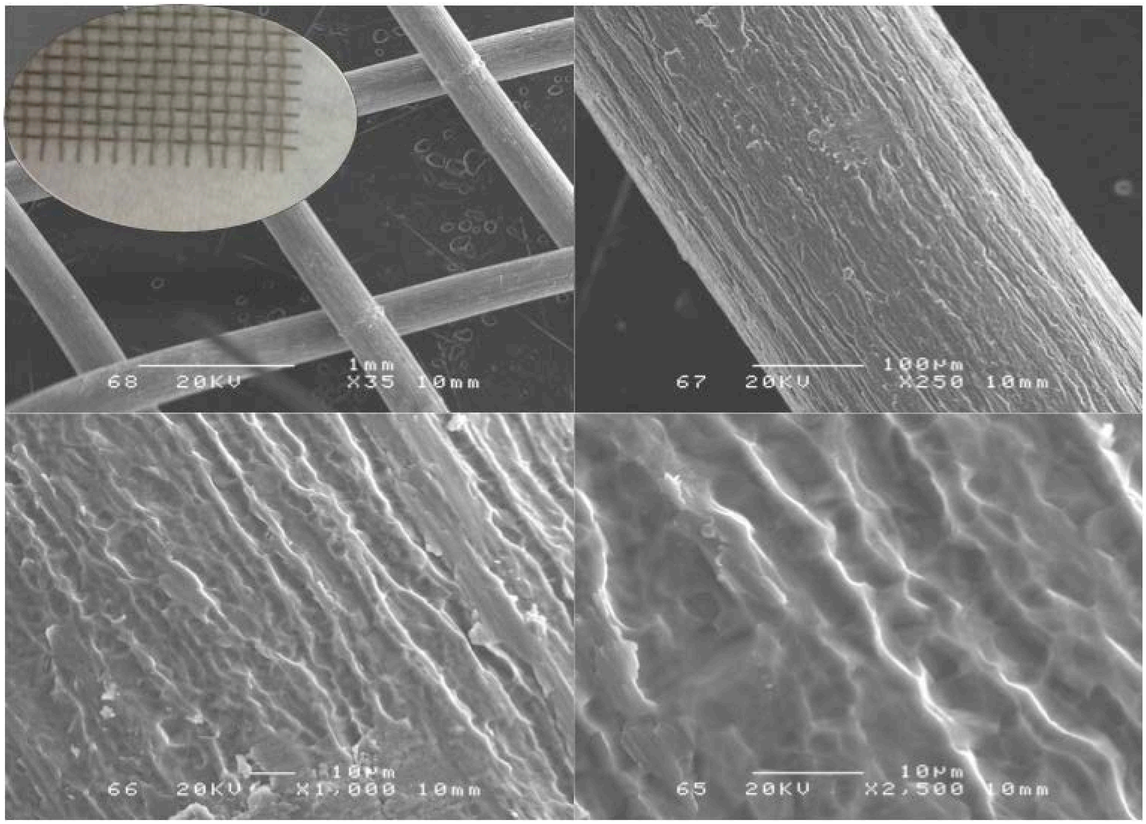

Figure S1. SEM micrographs at increasing magnifications of pristine Ti mesh. Inset at the top left figure represents a photograph of the Ti mesh.

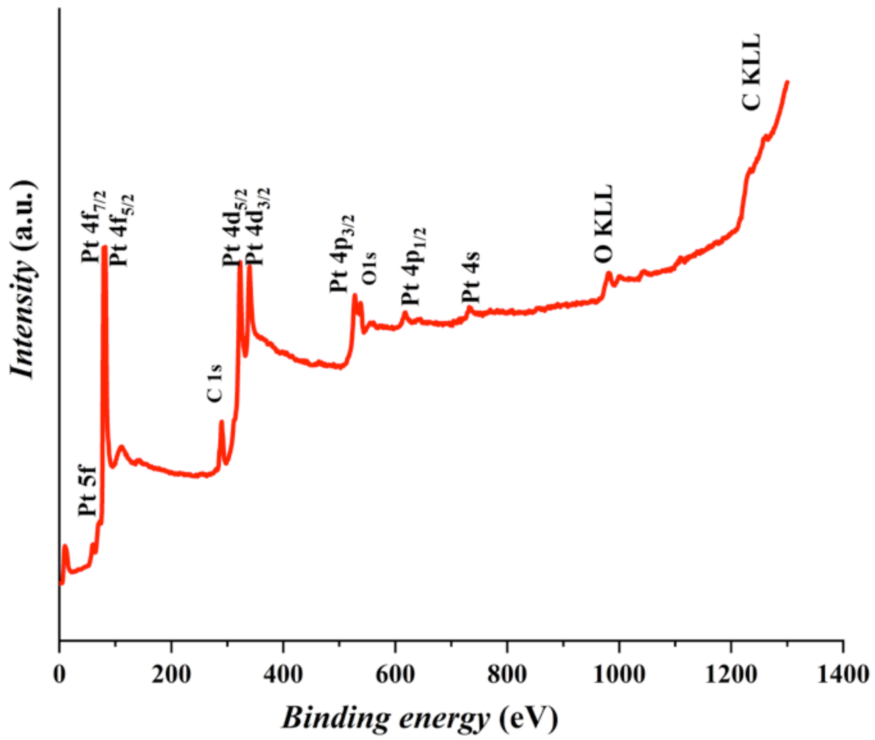

Figure S2. XPS survey scan of Pt film grown onto TiO_2 nanorods.

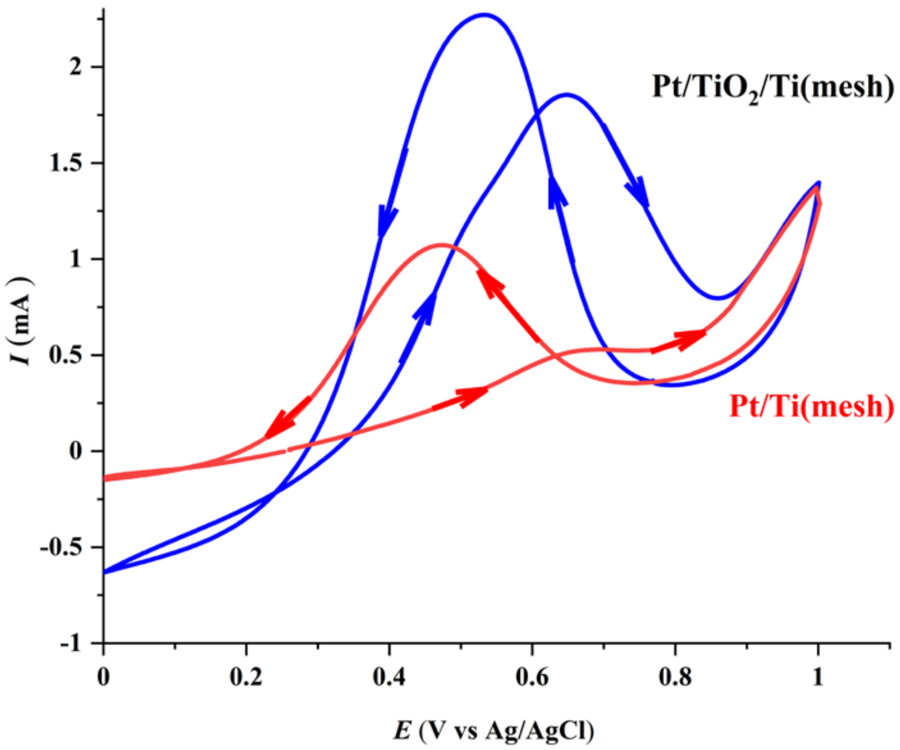

Figure S3. Cyclic voltammetry in 0.5 M H_2SO_4+ 1 M C_2H_5OH solution with a potential scan rate of 5 mV s^{-1}.
